# 

**Published:** 1911-01-15

**Authors:** 


CONTENTS

Event and Comment - - - - - - - - - - - - - - - - - - 1

Dental Anatomy, - - - - - - - - - *By N. S. Hoff, D.D.S.* 4

Can Pyorrhea Alveolaris be Cured? If so, what Constitutes a Cure? - - - - - - - *By Austin F. James, D.D.S.* 11

Experiences with Casting Metals, - *By C. N. Thompson, D.D.S.* 16

Indents - - - - - - - - - - - - - - - - - - - - - - 30

Announcements - - - - - - - - - - - - - - - - - - - - 47

